# Prevalence and Associated Factors of Depression among Pregnant Mothers Who Had Intimate Partner Violence during Pregnancy Attending Antenatal Care at Gondar University Hospital Northwest Ethiopia in 2020

**DOI:** 10.1155/2021/9965289

**Published:** 2021-06-03

**Authors:** Sileshi Ayele, Mekuriaw Alemayehu, Elfalet Fikadu, Gebrekidan Ewnetu Tarekegn

**Affiliations:** ^1^Department of Obstetrics and Gynecology, School of Medicine, College of Medicine and Health Sciences, University of Gondar, Gondar, Ethiopia; ^2^Department of Environmental and Occupational Health and Safety, College of Medicine and Health Sciences, University of Gondar, Gondar, Ethiopia; ^3^Department of Epidemiology and Biostatistics, Institute of Public Health, College of Medicine and Health Sciences, University of Gondar, Gondar, Ethiopia

## Abstract

**Background:**

Antenatal depression is the major obstetric problem that led to significant maternal and perinatal morbidity and mortality worldwide, especially in the third world. However, in Ethiopia this prevalence and association were not studied, as result, this study investigated the prevalence and associated factors of antenatal depression among pregnant mothers who had intimate partner violence during pregnancy. *Methodology*. An institution-based cross-sectional study was done among 409 pregnant mothers who had intimate partner violence during pregnancy from May to July 2019 at Gondar University Hospital. All pregnant mothers who came for ANC follow-up during the study period approached for screening of intimate partner violence during pregnancy using standard and validated screening method and instrument of the WHO multicountry study on women's health and domestic violence to evaluate intimate partner violence, and we use EPDS for the evaluation of antenatal depression validated in Ethiopia with a cut point of 13.

**Result:**

Prevalence of depression among pregnant mothers who had any form of intimate partner violence during pregnancy was 35%: physical abuse (AOR = 1.8; 95% CI: 1.19, 3.30), more than one type of abuse (AOR = 10.18; 95% CI: 7.10, 16.18), poor social support (AOR = 5.81; 95% CI: 1.12, 13.12), and pregnant mothers whose partner drunk for the past twelve months (AOR = 7.16; 95% CI: 183, 8.00) were significantly associated with antenatal depression.

**Conclusion:**

High prevalence of antenatal depression among pregnant mothers who had intimate partner violence during pregnancy was highly associated with physical abuse, more than one type of abuse, lack of social support, and partner of pregnant mothers who is a drunk. Hence, this is important to create a screening program and prevention strategy of intimate partner violence during pregnancy at the time of antenatal follow-up to prevent and early identify its morbidity and mortality.

## 1. Background

According to the World Health Organization (WHO), depressive disorders are characterized by sadness, loss of interest or pleasure, feeling of guilty or low self-worth, disturbed sleep or appetite, feeling of tiredness, and poor concentration. As it becomes severe, it can lead to individuals to suicidal ideation and suicidal attempt. The global burden of depression is rising dramatically; globally, around 322 million people were affected [1].

Globally, perinatal depression is a common problem and has significant devastating health outcomes, if unrecognized and untreated [2]. It also affects an estimated 10% to 20% of women during pregnancy. American College of Obstetrics and Gynecology recommended that an obstetrician and gynecologist and another health provider should perform at least once screening of pregnant women for perinatal depression and anxiety [2]. Although there are available known effective treatments of depression, only half of the affected people worldwide get treatment.

Many qualitative and quantitative studies in the world show that depression is highly associated with intimate partner violence (IPV). IPV according to the WHO is defined as any behavior within an intimate relationship that causes physical (such as slapping, hitting, kicking, and beating), psychological, (such as insults, belittling, constant humiliation, intimidation (e.g., destroying things), threats of harm, and threats to take away children), or sexual harm (including forced sexual intercourse and other forms of sexual coercion) to those in the relationship [3].

Globally, 30% of partnered women experienced physical and/or sexual IPV, one-third of women experienced partner or nonpartner violence in their lifetime [4]. Many systematic reviews and meta-analyses and other studies showed a high association and coexistence of depression and IPV. In Ethiopia, according to a hospital-based cross-sectional study among 450 pregnant women, 58.7% of them were a victim of one or more form of IPV [5].

The result of previous studies in the world revealed that several factors are associated with depression related to IPV. These include unplanned pregnancy [5, 6], partners alcohol use [6–8], family support during confinement [9], low income [8, 9], childhood sexual abuse [6], housewives [8], violence during pregnancy [5], and physical and emotional violence [10, 11].

Even though intimate partner violence is regarded important public health and human rights issue and a cause for a mother's depression during their pregnancy, to the best of our knowledge, there is limited evidence regarding the prevalence and associated depression-related factors with IPV among pregnant women in Ethiopia specifically in our setting.

Therefore, this study is aimed at determining the prevalence of depression and its associated factors in IPV among pregnant women. Conducting this study will help to alarm the ministry of health policymaker, to create preventive strategies and early identification of its bad health outcome particularly its mental health effect. Both IPV and depression neglected diseases in a developing country, especially in Ethiopia. This study will be also used as a source for a further study on the association of IPV and depression and their maternal and fetal outcome.

## 2. Methods and Materials

### 2.1. Study Design and Setting

An institution-based cross-sectional study design was done among pregnant women who had IPV during pregnancy having ANC follow-up at Gondar University Hospital from May 01 to July 30, 2019. Gondar University Hospital is found in the ancient and historic town of Gondar, northwest Ethiopia, 741 km from Addis Ababa, the capital city of Ethiopia. It is one of the biggest tertiary level referrals and teaching hospitals in the Amhara Regional State and provides preventive and curative services to over 5 million inhabitants in the catchment area. The hospital consists of 4 operating rooms, 4 intensive care units, and 13 wards with 327 beds. The antenatal care clinic is one of the departments which provide services to 30–40 pregnant women coming from Gondar town and the nearby districts per day. The hospital also serves as a research center and provides practical training to medicine and health science students.

### 2.2. Inclusion and Exclusion Criteria

All pregnant women who had IPV during pregnancy having ANC follow-up at GUH during the study period were included in the study, and pregnant women who need emergency intervention were excluded from the study.

### 2.3. Population, Sample Size Determination, and Sampling Procedure

The source populations for this study were all pregnant mothers who had ANC follow-up at GUH and all pregnant mothers who had IPV during pregnancy who have antenatal care at GUH during the study period (from May 01 to July 30, 2019). The required sample size was estimated using a single population formula: *n* = (*Za*/2)^2^(*P*(1 − *P*)/*d*^2^); by considering the prevalence of antenatal depression and intimate partner violence based on a similar study and considering a confidence level of 95% and a marginal error of 5%, the required sample size was 372, and by adding 10% of nonresponse rate, the final sample size was 409. All pregnant mothers who came for ANC follow-up during the study period were selected after the screening was done for IPV during pregnancy by using the WHO multicountry study on women's health and domestic violence to evaluate intimate partner violence with a 98.9% of response rate. Those who had any form of IPV during pregnancy were selected consecutively till the required sample size was reached.

### 2.4. Study Variables

The dependent variable was antenatal depression related to IPV. It was dichotomized as “Yes” if a woman had experienced depression related to IPV and “No” if a woman did not experience antenatal depression related to IPV.

Independent variables for this study included age, party, religion, residency, educational status, occupation, marital status, partner alcohol use, partner smoking, social support, the complication in a previous pregnancy (hemorrhage, infection, preterm birth, and premature rupture of membrane), medical illness (DM, HIV, and HTN), and partner feeling about current pregnancy.

### 2.5. Data Collection Instrument

The Edinburgh Postnatal Depression Scale (EPDS) was used to detect depressive symptoms. The EPDS is a 10-item questionnaire, scored from 0 up to 3 (higher score indicating more depressive symptoms). This has been validated for detecting depression in antepartum and postpartum samples in many countries like other similar studies conducted abroad and in Ethiopia. EPDS was validated in Ethiopia with the local language as a cutoff point of 13 [12, 13]. Therefore, we use an EPDS value of 13 and above to identify pregnant mothers with depressive symptoms. Those pregnant women who scored 13 and above were categorized as depressed women while pregnant women who score below 13 were considered nondepressed women [14].

WHO Woman Abuse Screening Tool (WAST) was used to screen pregnant women for intimate partner violence. Scores on the WAST are computed based on a criterion cutoff score of 1, which involves assigning a score of 1 to the most extreme positive responses for each of the items. The questions are used to gain a more complete assessment of the abuse by asking the respondent to rate the frequency of various feelings and experiences on a scale from 1 (often) to 3 (never). The WAST items are recoded and summed to calculate the overall score.

Intimate partner violence according to the WHO is defined as any behavior within an intimate relationship that causes physical, psychological, and sexual harm [4].

### 2.6. Data Collection Procedures and Data Quality Control

A structured questionnaire was administered by the interviewer for each participant in a private room. Before data collection began, the pilot study was conducted on 5% of sample size on similar study participants out of study area. Based on the results, corrections and modifications were made to the questionnaire before we applied to the study area. The face and content validity of the tool were examined by the researcher and healthcare professionals. The internal consistency of the tool was assessed, and Cronbach's alpha was computed (*α* ≥ 0.75), which was acceptable for this population. Data were collected by three midwife nurses who have experience in the data collecting process and a medical doctor who supervised the data collectors. The data collectors and supervisor take one-day training about the data collection process; the filled sheet was checked for completeness and consistency by the supervisor and principal investigator to ensure the quality of the data. Additional recheck and crosscheck of data entry were made; meanwhile, any doubts in the filled sheet are clarified for the data collector.

### 2.7. Data Processing and Analysis

Data was extracted from pregnant mothers using a standard questionnaire tool. Data were entered using Epi info version 7.1 and analyzed using SPSS software version 20. Descriptive statistics analysis was used to summarize the result. Since our outcome variable has two responses, we employed binary logistics regression. Each variable was evaluated independently in a bivariable analysis, and association was determined using COR at a 95% confidence interval. All variables associated with depression at a *P* value < 0.2 on the bivariable analysis were entered into a multivariable binary logistics regression analysis to control confounders. Variables with *P* value < 0.05 in the multivariable analysis reported as statistically significant variables that are associated with depression.

### 2.8. Ethical Clearance

Ethical clearance was obtained from the institutional review board of the College of Medicine and Health Sciences University of Gondar, and permission was obtained from the Department of Obstetrics and Gynecology. Informed consent was obtained from each pregnant mother who was participated in the study. Privacy and confidentiality are also maintained throughout the data collection.

## 3. Result

### 3.1. Sociodemographic Characteristics of the Participant

A total of 860 pregnant mothers were included with a response rate of 98.9%. A total of 409 (47.6%) pregnant mothers were found to encounter at least one form of IPV. Most of the participants were age between 25 and 34 (68.9%). The majority (95.6%) of participants were coming from urban. Three-fourth (75.9%) of pregnant mothers were of Orthodox religion. Nearly one-third (36.9%) of participants had attended educational level of college and above, whereas nine percent (*n* = 38) of the participants were uneducated. The majority of pregnant women were housewives (42.8%), and nearly one-third (28.4%) of the participants were government employees (Table 1).

### 3.2. Reproductive Characteristics of the Participants

The majority of pregnant women were of gestational age at the second trimester (40.6%) and third trimester (40.1%). Among the participants, two hundred fifty-four (62.1%) were having previous pregnancy, and of those pregnant mothers who had previous experience of pregnancy, one hundred one (39.8%) have one live baby, and a quarter of them have two children. Two hundred eleven (83.7%) pregnant women were having previous antenatal care. The majority of pregnant mothers (367 (89.7%)) have planned pregnancies. Nearly a quarter of them (23.6%) were having previous pregnancy complications during pregnancy and labor and delivery. Nearly two-thirds (64%) of our participants gave birth vaginally in their previous pregnancy, whereas a quarter of participant's mode of delivery was via cesarean section. Twenty-four (5.9%) of pregnant mothers were having chronic illnesses, and of this, most commonly identified (66.7%) type of illness was hypertension (Table 2).

### 3.3. Partner's Characteristics of Pregnant Mothers

Nearly half of partners of pregnant mothers were government employees. Almost one-fifth (19.6%) of partners of pregnant mothers were equally educated as their wife, and 268 (65.5%) of partners of pregnant mothers were more educated than their wife. More than half (52.3%) of the partners of a pregnant mother utilized alcohol sometimes, and nearly one-third (35.9%) of partners of spouses were drunk sometimes over the past twelve months. Three hundred forty-nine (85.5%) of partners of pregnant mothers never use chat, and only sixteen (3.9%) of partners use chat on a daily basis (Table 3).

### 3.4. Type of Intimate Partner Violence Encountered by Participants during Pregnancy

The prevalence of depressive episodes among pregnant mothers who had any form of intimate partner violence during pregnancy was 35%.

**Figure 1:**
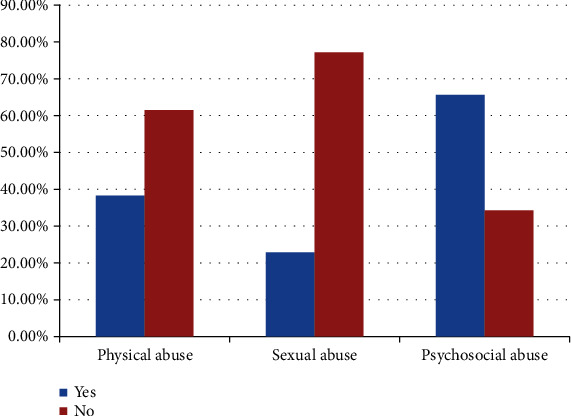
Type of intimate partner violence encountered by participants during pregnancy who had ANC follow-up at Gondar University Hospital, Northwest Ethiopia, 2019 (*n* = 409).

The most common type of intimate partner violence among participants was emotional violence (two hundred sixty-nine (65.8%)), physical violence (one hundred fifty-seven (38.4%)), and sexual violence (ninety-three (22.7%)) of pregnant mothers. A quarter (24.7%) of pregnant mothers had more than one type of intimate partner violence (Figure 1).

### 3.5. Factors Associated with Depression in Pregnant Mothers Who Had Intimate Partner Violence during Pregnancy

Variables such as residency, marital status, social support, partner use of alcohol, planned pregnancy, educational level of pregnant mothers, current pregnancy complication, partner feeling about the current pregnancy, partner use of alcohol, partner feeling about the current pregnancy, physical abuse, emotional abuse, sexual abuse, and more than one type of abuse were entered to multivariable analysis.

The depressive episode was more prevalent in pregnant mothers who had physical abuse during pregnancy as compared to those who had never experienced physical abuse during pregnancy with an adjusted odds ratio (AOR) = 1.8 (95% CI: 1.19, 3.30). Mothers who had more than one type of intimate partner violence during pregnancy had much more reported depressive episodes than those who had one or no intimate partner violence during pregnancy (AOR: 10.18; 95% CI: 7.10, 16.18). The depressive episode was identified more in pregnant mothers who had poor social support than pregnant mothers who had good social support (AOR: 5.81; 95% CI: 1.12, 13.12). Pregnant mothers whose partners drunk for the past twelve months were reported to have significantly high depressive episodes than mothers whose partners never drunk (AOR: 7.16; 95% CI: 183, 8.00) (Table 4).

## 4. Discussion

The result demonstrated in this study was much higher than that in the study done in Bangladesh where the prevalence of depression is 16.8% [15]. This low prevalence can be explained by the study population not specific to pregnant mother, and the overall prevalence of depression in this study was much lower than the prevalence of depression in pregnant mothers of Ethiopia which were 25.8% in meta-analysis and systematic review done by Mersha et al. [16]. However, this study is consistent with what has been found in Slovenia where the prevalence of depression was 36.9% among adult women who had intimate partner violence exposure [17] and in Bangladesh where the prevalence of depression was 35.2% among pregnant mothers [18]. This study shows the prevalence of any form of IPV during pregnancy is 47.6%, and the commonest type of abuse during pregnancy is emotional violence (65%) followed by physical abuse (38.4%) and sexual violence (22.7%) among pregnant mothers. A quarter of pregnant mothers experienced more than one type of intimate partner violence during pregnancy (Table 3). This finding shows the lower overall prevalence of IPV when we compared to a study done in a similar study area done by Belay et al. [6], where they found the prevalence of IPV was 58.7%. This difference may be due to the sociodemographic distribution differences among participants, like housewife, place of residency, and educational level in which they are associated with IPV during pregnancy. However, the pattern of IPV distribution among participants was similar to this study which was done in Ethiopia at the same study area done by Fekadu et al. [5]. On the other hand, we compared a study done in Bangladesh by physical IPV which was 66.7%, followed by sexual and emotional IPV [18]. This is more likely due to their study which included IPV that occurs before, during, and after pregnancy and also when we compared our people reporting physical and sexual violence who are more sensitive and ashamed of it compared to those with emotional violence to expose it to the public.

We obtain a significant association of physical abuse with depression episodes during pregnancy. This is because of a pregnant mother who experienced physical abuse more likely to also experience another type of abuse. This result is in line with the previous study done in Sweden, Japan, Latin America, and many other countries [10, 19, 20]. Contrary to the finding of significant association of physical abuse with depression episode, we did not find a significant association of sexual and emotional abuse with depression episode in pregnancy. However, this result is consistent with what has been found in a previous study in Ethiopian rural women [21]. This may have a different explanation; one explanation is the misunderstanding in the community where they consider the husband has executive power over his wife and the right to have sex with his wife without her consent; another one is lack of police which creates awareness between the community about intimate partner violence and its consequence; hence, the women did not considered it as violence and they did not yet understand its mental health effect till its hidden problems were discovered, especially in Ethiopia. Unlike another study, we were not able to investigate the relationship between the severity of physical abuse and its effect on antenatal depression.

This study most importantly demonstrates that poor social support was significantly associated with a depressive episode during pregnancy, and there was a similar result demonstrated by Ahmad et al. [22]. This is because social support exhibits a protective effect by buffering the effect of stress events on the emotional wellbeing of pregnant mothers and it also improves health-seeking behavior and overall improves maternal and fetal outcomes [23]. Therefore, pregnant mothers who have poor social support are more at risk of depression than pregnant mothers who have good social support.

The other basic result of this study is the partner of a pregnant mother who had a drunken significant association with a depressive episode during pregnancy. Hence, this study gives the insight to have a prevention strategy plan by involving both partners in antenatal care. This finding is in line with the finding of a study in Victoria, Australia, that hazardous drinkers are highly associated with depression. This is because women whose partners are drunk experience more violence than those whose partner never drunk and those partners who drunk are less responsible in the family; hence, women suffer more from depression [24, 25].

### 4.1. Strength and Limitations of the Study

The strength of this study is we used a standard and validated screening method and instrument of WHO multicountry study on women's health and domestic violence to evaluate intimate partner violence, and we used EPDS for the evaluation of antenatal depression which validated in Ethiopia with local language. One apparent limitation of this study was that we employed a cross-sectional study design that cannot tell as to whether causal relationships are present or not. In addition to this because of lack of time and budget, we were not able to further investigate maternal and fetal outcomes of depression.

## 5. Conclusion

The prevalence of depression among pregnant mothers who have intimate partner violence during pregnancy was very high. Antenatal depression is significantly associated with physical abuse, more than one type of abuse, lack of social support, and drunk partner of pregnant mothers. The Ministry of Health and policymakers should create a screening strategy program in antenatal care to prevent and identify early adverse mental health outcomes of intimate partner violence.

## Figures and Tables

**Table 1:** Sociodemographic and reproductive characteristics of pregnant mothers who had ANC follow-up at Gondar University Hospital, Northwest Ethiopia, 2019 (*n* = 409).

Variables	Frequency (*n*)	Percentage (%)
Age (years)		
15-24	78	19.1
25-34	282	68.9
>34	49	12
Religion		
Orthodox	325	79.5
Muslim	78	19.1
Protestant	6	1.5
Residency		
Urban	391	95.6
Rural	18	4.4
Marital status		
Single	20	4.9
Married	389	95.1
Level of educational		
Uneducated	38	9.3
Grades 1-6	78	19.1
Grades 7-12	142	34.7
College and above	151	36.9
Current occupation		
Farmer	6	1.6
Merchant	63	15.4
Student	24	5.9
Housewife	175	42.8
Government employer	116	28.4
Others	25	6.1

**Table 2:** Reproductive characteristics of pregnant mothers who had ANC follow-up at Gondar University Hospital, Northwest Ethiopia, 2019 (*n* = 409).

Variables	Frequency	Percentage
Gestational age		
1^st^ trimester	79	19.3
2^nd^ trimester	166	40.6
3^rd^ trimester	164	40.1
Previous pregnancy		
Yes	254	62.1
No	155	37.9
Numbers of living children		
None	31	12.2
One	101	39.8
Two	64	25.2
Three or more	58	22.8
Previous stillbirths		
Yes	20	7.9
No	234	92.1
Number of previous abortions		
None	205	80.7
One	44	17.3
Two	5	2
Method of last abortion		
Spontaneous	44	89.8
Induced	5	10.2
Is pregnancy planned?		
Yes	367	89.7
No	42	10.3
Previous pregnancy follow-up		
Yes	211	83.7
No	41	16.3
Complication during pregnancy		
Yes	59	23.6
No	191	76.6
Current pregnancy complication		
Yes	12 (2.9%)	2.9
No	397 (97.1%)	97.1
Mode of last delivery		
Vaginal	151	64
Forceps	23	9.7
Cesarean	62	26.3
Chronic medical illness		
Yes	24	5.9
No	385	94.1
Type of medical illness		
HIV	2	8.3
Diabetes	6	25
Hypertension	16	66.7
Social support		
Good	381	93.2
Poor	28	6.8

**Table 3:** Characteristics of partners of pregnant mothers who had ANC follow-up at Gondar University Hospital, Northwest Ethiopia, 2019 (*n* = 409).

Variables	Frequency	Percent
Occupation		
Farmer	35	8.6
Merchant	133	32.5
Student	2	5
Employer	194	47.4
Others	45	11
Level of education		
Equal educational level	80	19.6
More educated than his wife	268	65.5
Less educated than his wife	61	14.9
Feeling about the current pregnancy		
Happy	385	94.1
Unhappy	24	5.9
Alcohol use		
Often	42	10.3
Sometimes	214	52.3
Never	153	37.4
How many times he was drunk the past 12 months		
Sometimes	92	35.9
Never	164	64.1
Chat use		
Often	16	3.9
Sometimes	44	10.8
Never	349	85.3
Cigarette use		
Often	8	1.9
Sometimes	6	1.5
Never	395	96.6

**Table 4:** Bivariable and multivariable binary logistic regression analysis of factors associated with antenatal depression among pregnant mothers who had intimate partner violence who had antenatal care at Gondar University Hospital, Northwest Ethiopia, 2019 (*n* = 409).

Variables	Depression		COR (95% CI)	AOR (95% CI)
	Yes	No		
Age (years)				
15-24	37	41	1.425 (0.689-2.946)	
25-34	87	195	0.704 (0.376-1.320)	
>34	19	30	1	
Religion				
Orthodox	109	216	0.252 (0.046-1.399)	
Muslim	30	48	0.313 (0.054-1.812)	
Protestant	4	2	1	
Residency				
Urban	127	264	1	
Rural	16	2	16.63 (3.77-73.43)	
Marital status				
Single	13	7	3.700 (1.44-9.49)	
Married	130	259	1	
Level of educational				
Illiterates	21	17	4.25 (2.02-8.95)	2.34 (0.46-11.64)
Grades 1-6	42	36	4.02 (2.23-7.22)	0.59 (0.08-4.23)
Grades 7-12	46	96	1.65 (0.98-2.77)	0.45 (1.06-1.91)
College and above	34	117	1	
Gestational age				
1^st^ trimester	29	50	1.032 (0.591 1.803)	
2^nd^ trimester	55	111	0.882 (0.560-1.389)	
3^rd^ trimester	59	105	1	
Numbers of living children				
None	10	21	0.675 (0.270-1.687)	
One	30	71	0.599 (0.305-1.175)	
Two	22	42	0.742 (0.356-1.547)	
Three or more	24	34	1	
Previous stillbirths				
Yes	10	10	2.079 (0.830-5.207)	
No	76	158	1	
Number of previous abortions				
None	67	138	1	
One	15	29	1.07 (0.54-2.12)	
Two	4	1	8.24 (0.90-0.75.15)	
Method of last abortion				
Spontaneous	17	30	1	
Induced	2	4	0.88 (0.15-5.33)	
Is pregnancy planned?				
Yes	121	246	1	1
No	22	20	2.24 (1.18-4.26)	1.28 (0.22-7.61)
Previous pregnancy follow-up				
Yes	74	137	1	
No	12	29	0.77 (0.36-1.59)	
Previous complication during pregnancy and labor				
Yes	28	31	2.071 (1.140-3.762)	2.32 (0.63-8.46)
No	58	133	1	1
Current pregnancy complication				
Yes	10	2	9.925 (2.144-45.946)	
No	133	264	1	
Mode of delivery				
Vaginal	38	113	1	
Forceps	14	9	4.63 (1.85-4.56)	2.90 (0.7.3-38.58)
Cesarean	28	34	2.45 (1.31-4.56)	3.90 (0.93-6.62)
Social support				
Good	125	256	1	1
Poor	18	10	3.69 (1.65-8.22)	5.81 (1.12-13.12)^∗∗^
Level of education				
Equal educational level	33	47	1	
More educated than his wife	91	177	0.73 (0.44-1.22)	
Less educated than his wife	19	42	0.64 (0.32-1.30)	
Feeling about the current pregnancy				
Happy	127	258	1	1
Unhappy	16	8	4.06 (1.69-9.75)	0.56 (0.05-4.17)
Alcohol use				
Often	26	46	3.780 (1.854-7.705)	
Sometimes	71	143	1.155 (0.738-1.807)	
Never	46	107	1	
Drunk the past 12 months				
Sometimes	57	35	5.089 (2.932-8.832)	7.16 (1.83-8.00)^∗∗^
Never	40	125	1	1
Chat use				
Often	6	10	1.145 (0.406-3.226)	
Sometimes	17	27	1.202 (0.630-2.292)	
Never	120	229	1	
Physical abuse				
Yes	80	77	3.117 (2.041-4.760)	1.81 (1.19-3.30)^∗∗^
No	63	189	1	1
Sexual abuse				
Yes	42	51	1.173 (1.094-2.810)	8.02 (0.71-8.92)
No	101	215	1	
Emotional abuse				
Yes	102	167	1.475 (0.950-2.289)	1.10 (0.29-4.12)
No	41	99	1	
More than one type of abuse				
Yes	74	27	9.49 (5.67-15.90)	10.18 (7.10-16.18)^∗∗^
No	69	239	1	1

1: reference; ^∗∗^*P* value < 0.05.

## Data Availability

The datasets used and/or analyzed during the current study are available from the corresponding author on reasonable request.

## Abbreviations

ACOG: American College of Obstetrics and Gynecology
ANC: Antenatal care
CI: Confidence Interval
CMHS: College of Medicine and Health Science
COR: Crude odds ratio
EDHS: Ethiopian demographic health survey
GUH: Gondar University Hospital
HMO: Health Maintenance Organization
IPV: Intimate partner violence
MDE: Major depression episode
OR: Odds ratio
PI: Principal investigator
PND: Postnatal depression
SPSS: Statistical Package for Social Scientists
UOG: University of Gondar
WHO: World Health Organization.
